# Medical School of a Northern University

**Published:** 1883-10

**Authors:** 


### MEDICAL SCHOOL OF A NORTHERN UNIVERSITY.

We return our thanks to the medical faculty of a great Northern medical school for their kind and handsomely expressed invitation to attend their one hundredth anniversary. We would indeed have enjoyed such an occasion, and the jaunt would have been of immense benefit to us by way of recreation, but unfortunately the affair came off in May last, and our invitation did not put in an appearance till the following October. Strange things will happen, as Mr. Arp would say. Our invitation to the bi-centennial will doubtless come in time. This gives us the opportunity to remark, that in five minutes it can be demonstrated to any Southern young man of ordinary sense that he loses both money and opportunity by leaving a better medical college for him at home, such for example as the Atlanta Medical Colleges, to study and graduate in one at the North, however hoary it may be in years.

